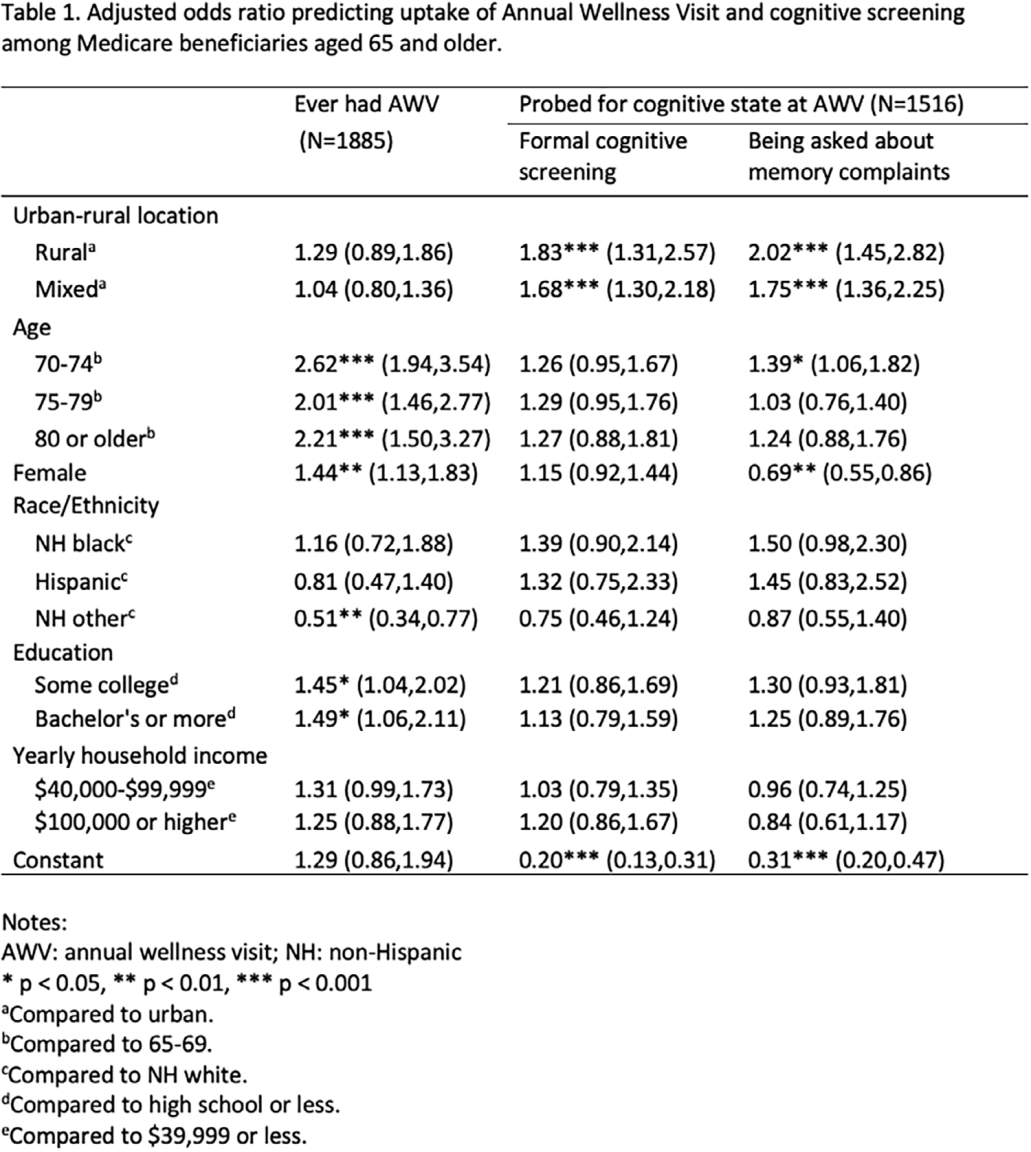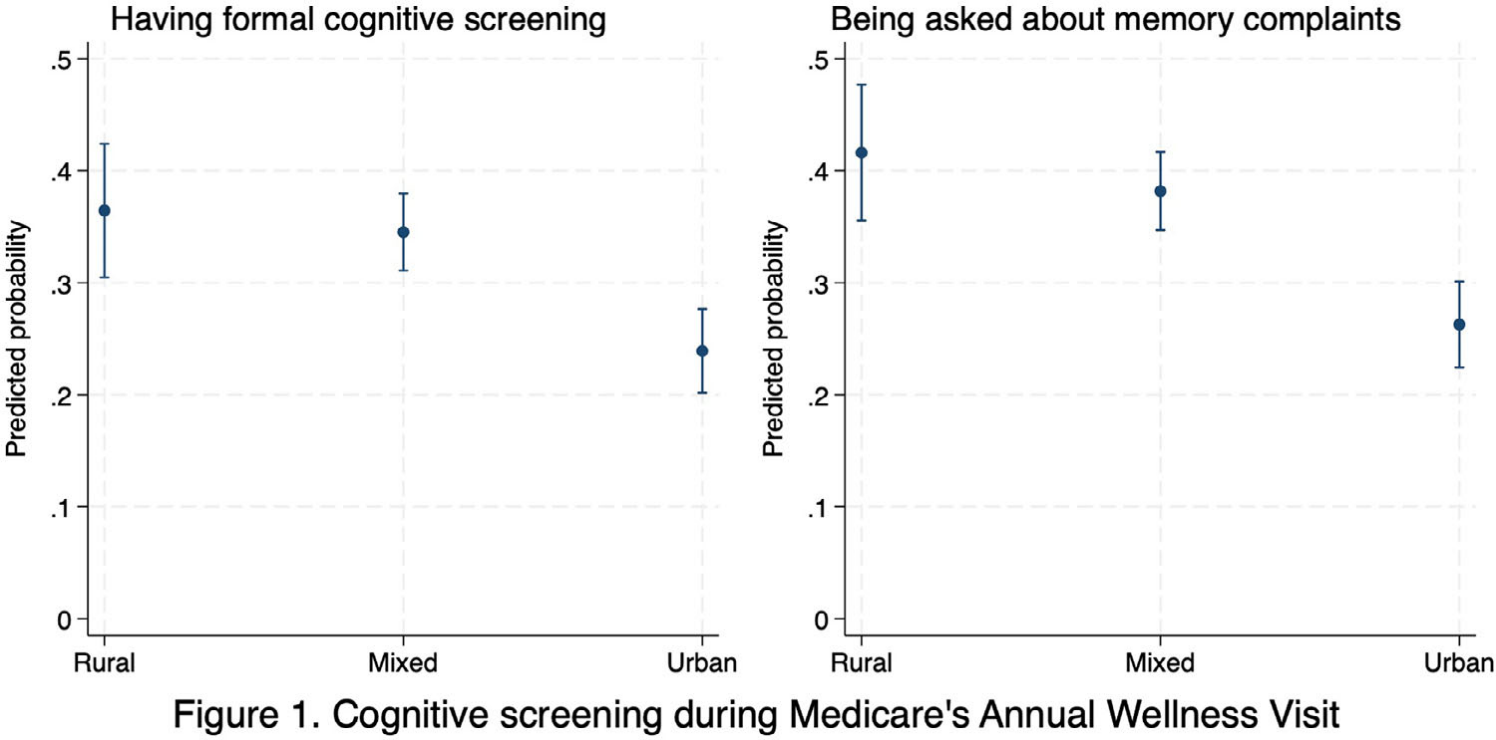# Uptake of Medicare’s Annual Wellness Visit and Cognitive Screening: Evidence of Urban‐Rural Disparity from a National Survey

**DOI:** 10.1002/alz.089530

**Published:** 2025-01-09

**Authors:** Ying Liu, Soeren Mattke

**Affiliations:** ^1^ University of Southern California, Los Angeles, CA USA

## Abstract

**Background:**

Early‐stage cognitive impairment remains severely under‐detected. In the U.S., Medicare covers an Annual Wellness Visit (AWV) that includes assessment of cognitive state, but it remains under‐utilized: only 55% of beneficiaries reported having had AWV, of whom only 26% were given a structured cognitive test in 2019. We obtained updated estimates and analyzed association with beneficiary characteristics.

**Method:**

We used a nationally representative survey, the Understanding America Study, to collect data on uptake of AWV and whether the visit included a formal cognitive test or simple questions about memory complaints in December 2023. The survey also collects data on demographics (age, sex, race/ethnicity, education, and yearly household income) and urbanicity of the participant’s residence. Logistic regression was used to predict a beneficiary having an AWV or been probed for cognitive state during the AWV using the beneficiary’s characteristics.

**Result:**

Table 1 shows the logistic regression results. Among 1,885 respondents, 81% reported having had an AWV, higher likelihood among older, female, and higher education beneficiaries. During AWV, 51% were probed for their cognitive condition: 31% with formal screening and 35% being asked about subjective memory complaint.

Despite no urban‐rural difference in AWV’s uptake (compared to urban residents, Odds Ratio [OR] = 1.29 for rural and OR = 1.04 for mixed, p>0.05), conditional on the uptake, residents of rural (OR = 1.83, p<0.001) and mixed (OR = 1.68, p<0.001) areas were more likely to undergo formal screening than their urban counterparts, with adjusted probabilities of 36% for rural, 34% for mixed, and 24% for urban (Figure 1). Results are similar on being probed about memory complaints.

**Conclusion:**

AWV uptake is increasing, despite a lesser increase in cognitive screening. Female, older, and higher educated beneficiaries are more likely to use the AWV. The unexpected finding of urban residents being less likely to be probed for cognitive impairment deserves further investigation. Potential explanations include clinicians’ awareness of higher disease burden in rural populations, and greater familiarity with their patients. Also, limited access to specialists could cause clinicians to offer a greater range of diagnostic services themselves and become more experienced in cognitive testing.